# The Effects of Artificial Intelligence in Quality Assessment of Outpatient Mental Health Services on Human Auditors

**DOI:** 10.1055/a-2961-0302

**Published:** 2026-09-30

**Authors:** Hayden T. McLeod, Joshua L. Cohen, Dena M. Bravata, Roma M. Bajaria, Kelvin Tran, Neha P. Chaudhary, Nicole M. Benson, Jeff Gould

**Affiliations:** 1 TherapyMatch, Inc (dba Headway)New YorkNew YorkUnited States; 2 Center for Primary Care and Outcomes Research 6429 Stanford UniversitySan MateoCaliforniaUnited States; 3 Harvard Medical School 2348 Massachusetts General HospitalBostonMassachusettsUnited States; 4 Harvard Medical School 24472 McLean HospitalBelmontMassachusettsUnited States; 5 8785 University of CaliforniaSan FranciscoCaliforniaUnited States

**Keywords:** system improvement, quality, clinical decision support, artificial intelligence, electronic health records and systems, psychiatry

## Abstract

**Background** The use of large language model (LLM)-based artificial intelligence (AI) to assess the quality of outpatient mental health documentation represents an understudied application. Critical questions remain about the effects of exposing human auditors to AI-generated recommendations on auditor efficiency, agreement, and patterns of decision-making.

**Objectives** The objective of this study is to examine how exposure to LLM-generated quality assessments influences auditor performance during reviews of documents of outpatient mental health intake sessions.

**Methods** We conducted a pre–post observational evaluation of 10 trained human auditors who reviewed outpatient mental health intake notes and assigned quality ratings using a standardized 13-item chart audit rubric before and after implementation of LLM support. The AI system used Anthropic Claude Sonnet 4 with a fixed chain-prompting strategy. We assessed item-level pass frequency for each auditor across the pre-AI and post-AI periods, AI–human agreement (the alignment between auditor pass/fail determinations and AI-generated recommendations), and auditing time. The pre-AI period included 9,711 notes (126,243 item-level observations) and the post-AI period included 2,677 notes (34,801 item-level observations).

**Results** Following implementation of AI-supported auditing, the mean item-level pass rate increased by approximately 2%. The distribution of auditor-level changes was broad (range: −35 to +35%, standard deviation [SD]: 10%) indicating substantial heterogeneity in individual auditor responses to AI support. High auditor agreement with AI recommendations was associated with larger pass-rate changes for selected rubric items, with some auditors increasing and others decreasing pass rates relative to the pre-AI period. The average time for an auditor to review notes decreased from 8.77 min (SD: 9.64) per review during the pre-AI period to 8.06 min (SD: 10.28) during the post-AI period ( *p* = 0.002), an 8% improvement equivalent to 71 min saved per 100 notes reviewed.

**Conclusion** LLM-based AI tools may improve the efficiency of clinical quality assurance workflows, but exposure to AI recommendations may meaningfully change human auditor behavior.

## Background and Significance

Demand for high-quality mental health services continues to increase, prompting interest in informatics solutions that improve diagnostic accuracy, patient monitoring, treatment delivery, and quality assessment. 1 2 3 4 Artificial intelligence (AI) systems, including large language models (LLMs), have demonstrated promising performance for detecting, classifying, and predicting risk of mental health conditions; predicting treatment response, monitoring prognosis, and reducing clinical workloads. 1 4 However, concerns remain regarding algorithm bias, lack of standardized measures for AI-assisted assessment, limited external validation, and generalizability in routine clinical practice, especially in non-English language contexts. 4 5 These issues have led to calls for rigorous evaluations of AI-supported clinical decision-making in mental health informed by human–AI interaction and implementation science frameworks. 2

Within this broader landscape, the use of AI to assess outpatient mental health documentation quality represents an emerging but understudied application. Prior work has shown that automated measures can approximate human ratings of psychotherapy processes, 6 7 8 yet little is known about how trained human auditors respond when AI-generated recommendations are embedded in routine quality reviews. This gap is notable, given rising psychotherapy utilization, 9 10 substantial variability in adherence to evidence-based practices, 11 and the absence of widely adopted national benchmarks for psychotherapy quality. 12 Critical questions remain about whether exposure to AI recommendations improves auditor efficiency, changes agreement with model outputs, or introduces new patterns of decision-making within quality review workflows.

## Objectives

The objective of this study was to examine how exposure to LLM-generated quality assessments influences auditor performance during reviews of outpatient mental health intake evaluations. By examining changes in review time, AI–human agreement, and rating patterns, this study aims to inform the safe and effective integration of AI-supported quality assessment tools into mental health quality improvement workflows deployed at scale. This evaluation was reviewed using the STARE-HI framework for reporting evaluation studies in health informatics. 13 14

## Methods

### Study Design and Setting

We conducted a pre–post observational study within Headway’s large national mental health provider network that routinely audits intake evaluation notes for adherence to quality standards, documentation requirements, and evidence-based practice expectations and remediates providers not meeting quality standards through a process of education and reevaluation. Headway’s network of 80,000 providers collectively have more than one million patient visits per month. The study was a natural experiment: the organization introduced an LLM-based quality assessment tool into an existing audit workflow, and we compared auditor rating behavior before and after this implementation. The notes that were reviewed were of outpatient mental health intake evaluations, not ongoing psychotherapy progress notes. The 13-item rubric covered common elements expected in an initial comprehensive mental health evaluation, including presenting concerns, symptom–diagnosis alignment, substance use history, behavioral health history, trauma history, self-harm and harm-to-others history, mental status examination, suicidal ideation (SI) and homicidal ideation (HI), safety planning, other high-risk scenarios, and evidence-based treatment planning. The rubric was primarily designed to assess clinical documentation quality and safety-relevant content; it was not used to determine payment denial. To evaluate the effects of AI assistance on auditor performance, we compared rating behaviors before and after launching AI support. The preperiod, April to July 2025, represented routine auditing before the implementation and included 9,711 notes. The postperiod, August to September 2025, represented the same workflow after AI-generated quality assessments were introduced into the audit interface and included 2,677 notes.

### Large Language Model–Based Quality Assessment Tool

We used Claude Sonnet 4 (Anthropic), an LLM, with a fixed chained-prompting strategy, extended reasoning, and tool-use capabilities to guide the AI assessment. The LLM was not trained or fine-tuned on the study notes, the auditor sample, or identifiable clinical data; underlying model weights were not updated. Human experts developed the rubric definitions, prompt sequence, and output format.

For each note, the model was prompted to evaluate all 13 rubric items in a fixed standard order that did not vary across notes and did not branch based on prior responses. Within each item, the model first extracted candidate evidence passages from the note, then rendered a pass/fail determination conditioned on that extracted evidence and finally returned a structured item-level recommendation with a brief rationale citing the extracted text. Safety-relevant items, self-harm history, harm-to-others history, SI, HI, plan/intent/means, safety planning, and other high-risk scenarios, required additional evidence handling. The model was required to perform an explicit search of the note for documentation of each element and to record a null finding when no relevant text was present, before assigning any recommendation. The system was not allowed to cite evidence outside the note.

Expert human auditors rated whether the sample note satisfied or failed specific rubric criteria (e.g., completed mental status exam, elicited presence or absence of SI) and provided annotation. These annotated examples were used during prompt development and internal validation to assess whether the LLM outputs matched expert expectations; they were not used to train or fine-tune the model. The expert consensus responses used to qualify human auditors during training were separate from the notes included in the study analysis and were used to assess auditor competence, not to train the LLM. We did not conduct an independent adjudicated review of the full pre- and post-AI study samples, so the present analysis, evaluates changes in auditor behavior and AI–human agreement rather than definitive accuracy. The tool was run with fixed settings in routine use; repeated testing on a small internal sample during development was used to check for materially inconsistent outputs, and no recurrent hallucinated note content was observed. During the post-AI period, item-level pass/fail recommendations and the AI-generated rationale were shown to auditors in the audit interface. No AI outputs were available during the pre-AI period.

### Auditor Training and Workflow

Auditors were all licensed therapists with at least 3 years of clinical practice. They were recruited, screened for clinical competence through live case vignettes, and specifically trained for quality assessment. Auditors were part of the organization’s existing quality assurance team and conducted item-level reviews of intake evaluation notes. Their task was to determine whether each item, or quality criterion, passed (i.e., met rubric standards) or failed (i.e., did not meet standards; Fig. 1 ). As part of their training, auditors had to demonstrate at least 90% alignment on rubric items with expert consensus responses on a sample of training notes. The consensus responses served as a practical internal reference standard for auditor qualifications and calibration, but they were not used as an external gold standard for the study outcomes. Auditor performance is reviewed quarterly to ensure continued interrater reliability. Auditors were expected to complete high-quality reviews; they were not assigned payment denial targets. Routine operational monitoring included review volume and timeliness, but there were no incentives to increase fail rates.

**Fig. 1:**
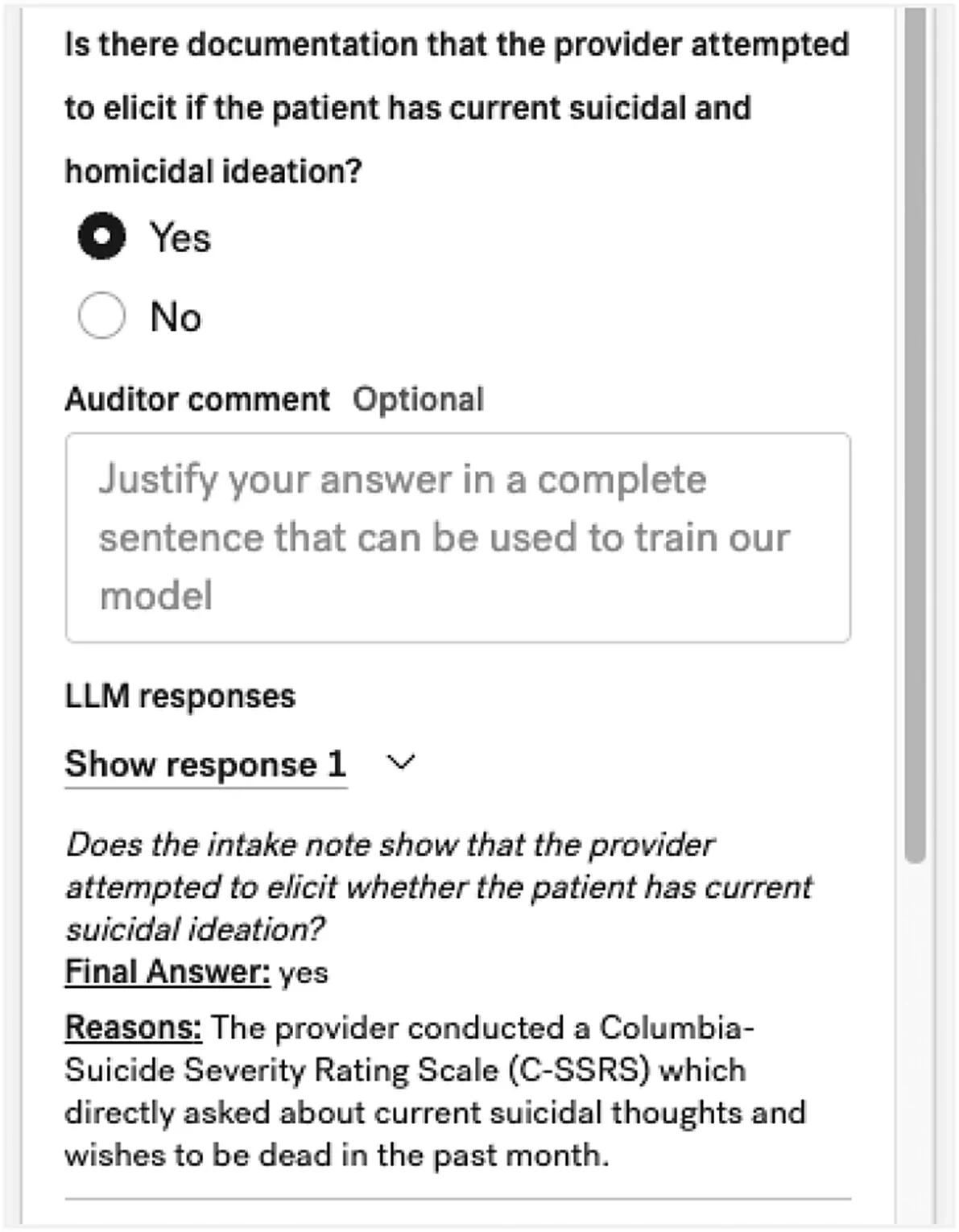
Screenshot of auditor tool with LLM responses. The figure displays the tool used by auditors to rate provider notes against the quality rubric. It also shows the type of response from the LLM for the same rubric item. LLM, large language model.

In the pre-AI period, auditors reviewed intake evaluation notes in accordance with the rubric, selecting pass or fail for each item without AI input. In the post-AI period, auditors saw the AI-predicted answer (i.e., pass or fail) and an AI-generated explanation supporting the recommendation within the audit interface before finalizing their own determination. Thus, auditors were not blinded to AI recommendations during the post-AI period. The workflow was rolled out as part of routine quality improvement rather than as a randomized trial.

### Measures

The primary outcome was the item-level pass frequency for each auditor across the pre-AI and post-AI periods. Pre-AI reflected the proportion of rubric items an auditor passed before AI support was introduced. Post-AI reflected the proportion passed after AI-generated assessments were displayed. We calculated the change in pass frequency (post-AI minus pre-AI) as the difference in the number of items passed before and after implementation.

A key secondary outcome was AI–human agreement, defined as the alignment between auditor pass/fail determinations and AI-generated recommendations for the same note and rubric item. Because auditor ratings serve as the primary benchmark for evaluating AI performance, shifts in auditor behavior have direct implications for interpretation. Increased auditor agreement with AI recommendations could artificially inflate apparent AI performance even if the underlying pass/fail determinations were inaccurate. Conversely, alignment driven by improved auditor accuracy could be beneficial. Our primary concern was whether AI exposure changed human scoring patterns in ways that might produce inappropriate passing or failing scores, with potential clinical or operational consequences (e.g., triggering unnecessary remediation activities for clinicians).

We also examined auditing time, measured as the duration required to complete each intake evaluation note review. AI support may shorten review time, indicating potential efficiency gains. However, reductions in auditing time may also correspond to decreased attention to complex or high-stakes rubric items, particularly major-deficit items that are more likely to have patient safety or clinician remediation implications.

Additional secondary measures included changes in the distribution of pass/fail patterns across rubric domains and variability in auditor behavior over time.

### Statistical Analysis

Analyses were conducted using Python and the SciPy statistics package. We summarized item-level pass frequencies, AI–human agreement, and review time in the pre-AI and post-AI periods. The primary contrast of interest was the change in pass frequency associated with implementation of AI support. We compared pre-AI and post-AI review times using a two-sided Welch *t* test, because review-time distributions were unequal in size and variance. Auditor- and item-level changes were summarized descriptively and visually because observations were nested within auditors, notes, and rubric items. We did not conduct a prospective sample-size calculation, because this was a natural experiment using all available routine audit data before and after implementation. The pre-AI sample was larger because it covered 4 months of routine auditing, whereas the post-AI sample covered the first 2 months after rollout. Confidence intervals (CIs) were not estimated for all nested contrasts; future analyses should use hierarchical models that account for clustering by auditor, note, provider, and item. Statistical input was provided by study team members experienced in analysis of large clinical and operational datasets.

## Results

### Overall Changes in Pass Rates

Following implementation of AI-supported auditing, the mean item-level pass rate increased by approximately 2% (95% CI: 0.32–3.168%) compared with the preperiod ( Fig. 2 ). Although this shift in the overall mean was modest, the distribution of auditor-level changes was broad, ranging from –35 to +35%, with a standard deviation (SD) of approximate 10%, indicating substantial heterogeneity in individual auditor responses to AI support.

**Fig. 2:**
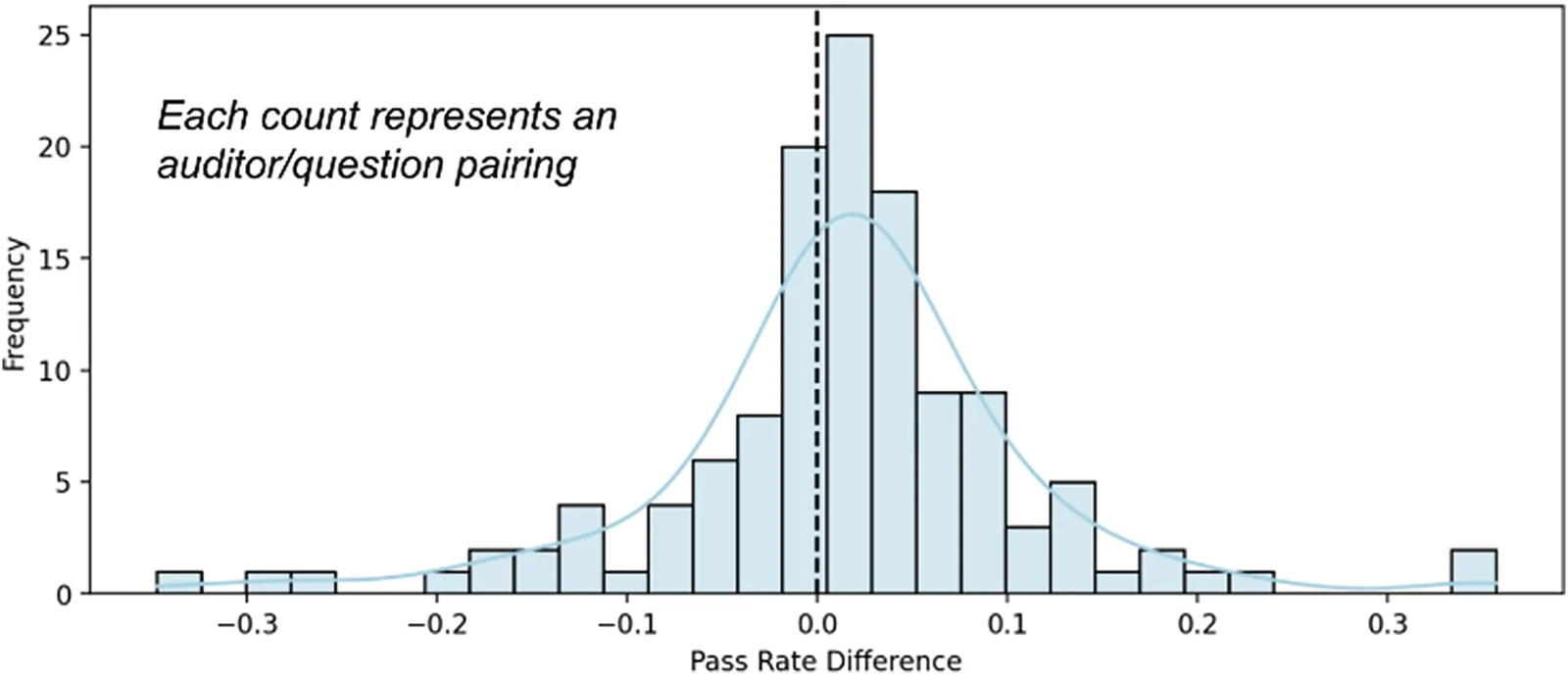
Distribution of auditor-level changes in item-level pass rates before and after AI implementation. The figure displays the change in pass frequency for each auditor between the pre-AI and post-AI periods. The overall mean increased by approximately 2%, with changes ranging from –35 to +35% (SD: ~10%). Variation suggests heterogeneous behavioral responses to AI support. AI, artificial intelligence; SD, standard deviation.

### Auditor-Level Changes

To determine whether observed changes in pass rates were driven by a small number of outlier auditors, we examined auditor-level distributions of pass-rate changes ( Fig. 3 ). Although several auditors had median shifts near the boundaries of the overall interquartile range, no single auditor exhibited an extreme or disproportionate change. This suggests that observed behavioral shifts were distributed broadly across the auditor pool, rather than concentrated among a few individuals.

**Fig. 3:**
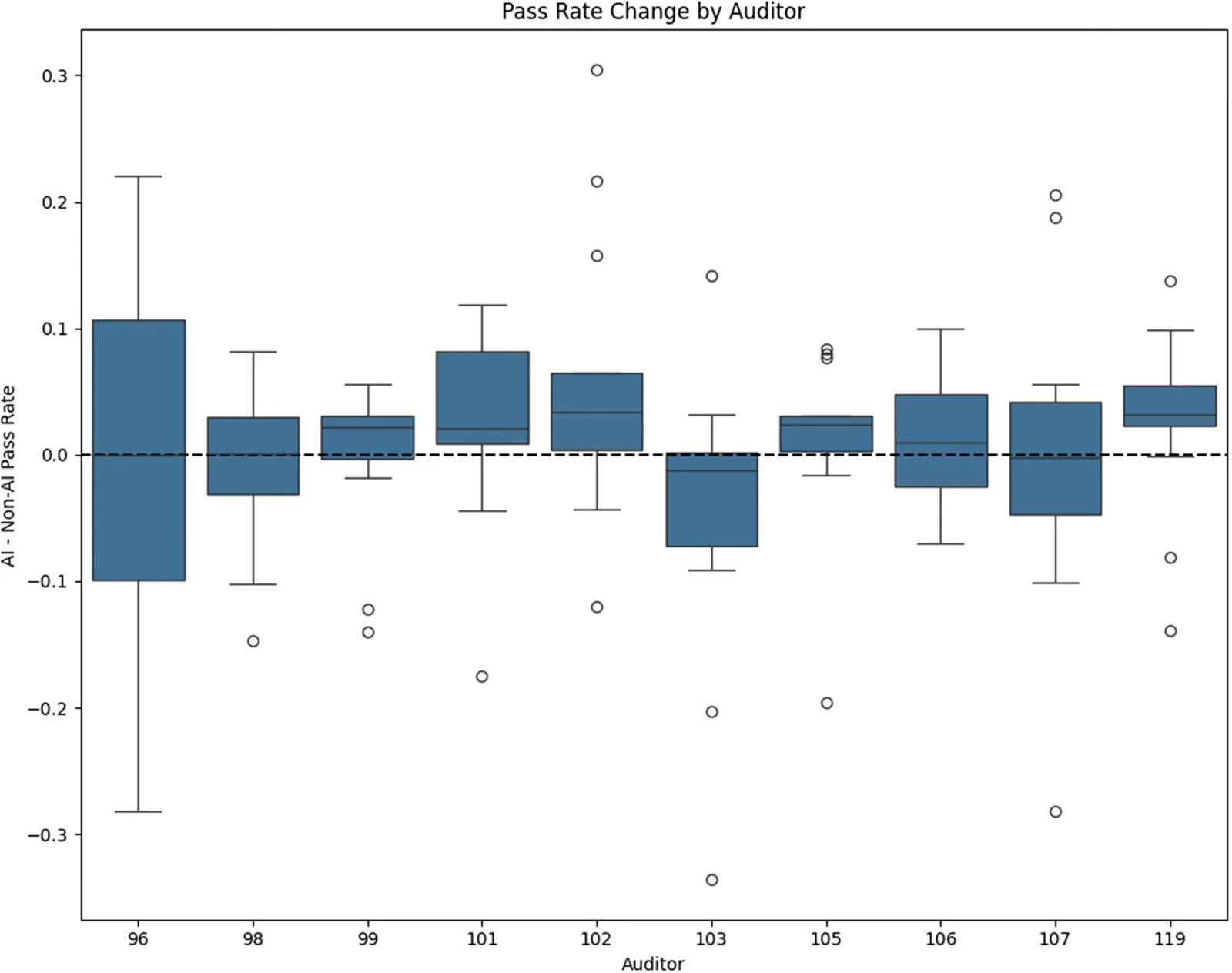
Distribution of auditor-level pass-rate changes by auditor. Boxplots summarize pass-rate shifts for each auditor. While some auditors show medians near the edge of the overall interquartile range, no single auditor exhibits extreme deviations, suggesting that observed effects reflect broader patterns rather than isolated behavior. AI, artificial intelligence.

### Artificial Intelligence–Human Agreement and Pass-Rate Shifts

We examined AI–human agreement rates versus changes in pass rates at the auditor and item level ( Fig. 4 ). Each dot represents the rate at which a single auditor agreed with AI recommendations and that auditor’s change in pass rate after implementation of AI for each rubric item. For items in which AI–human agreement rates ranged from 0 to 90%, the distribution of pass-rate changes remained narrow and centered near zero, indicating relatively stable behavior after integration of AI (green box). However, for the items with 90–100% AI agreement rates, the variance in pass-rate changes increased sharply, with large shifts observed in both positive and negative directions (yellow box). This pattern suggests that high AI–human agreement may be associated with behavioral influence, leading some auditors to increase and others to decrease pass rates relative to the pre-AI period.

**Fig. 4:**
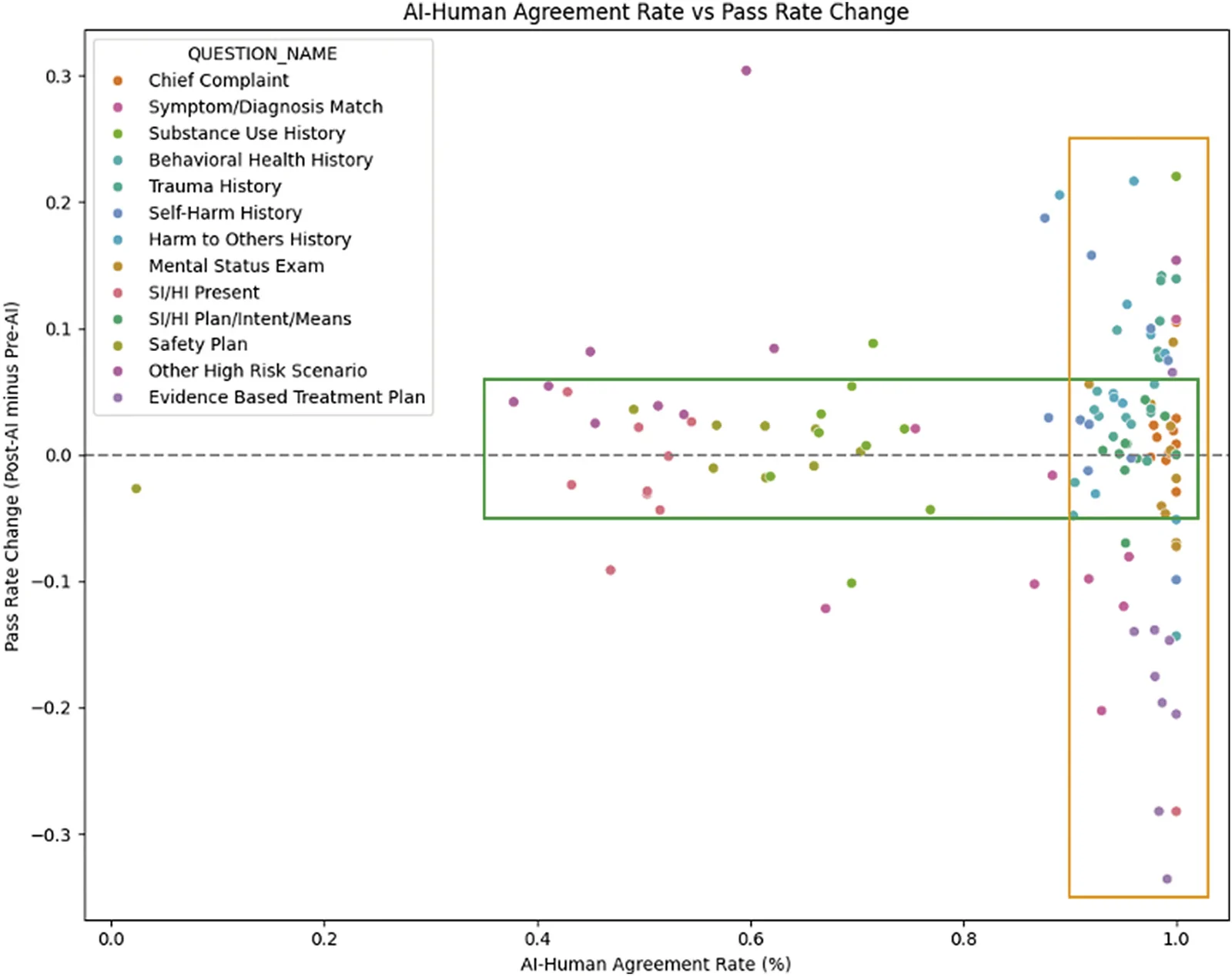
Scatterplot of AI–human agreement rate versus change in pass rate. Each point represents an item. The *x* -axis shows the AI–human agreement rate; the *y* -axis shows the corresponding change in pass rate. Items with AI–human agreement <90% cluster near-zero change, whereas those with >90% agreement show markedly increased variance, suggesting potential influence of AI predictions on auditor behavior. AI, artificial intelligence.

Additionally, individual rubric items appeared to cluster into lower agreement-stable behavior and higher agreement-behavior change groups, indicating that auditor behavioral change was concentrated in specific items that had high AI–human agreement across all auditors.

### Item-Level Changes

Item-level analyses revealed several items with notable deviations from typical behavior. The boxplots in Fig. 5 depict the change in pass rates and the rate at which auditors agreed with AI recommendations. Most items exhibited only small changes that were centered at 0%, indicating auditor behavior was generally unchanged after AI introduction. However, several items demonstrated meaningful deviations from the norm, with significant shifts in pass rates and very high AI–human agreement. These patterns may indicate AI influence on auditor decision-making. For example, the Evidence Based Treatment Plan item showed a substantial decrease in pass rate while displaying near-universal AI–human agreement, suggesting strong auditor alignment with AI outputs. Trauma History, Harm to Others History, and Self-Harm History demonstrated notable increases in pass rates accompanied by high AI–human agreement. Because there was no independent adjudicated gold standard, these changes should not be interpreted as proof that either auditors or the AI were more accurate; rather, they identify items requiring targeted calibration and validation.

**Fig. 5:**
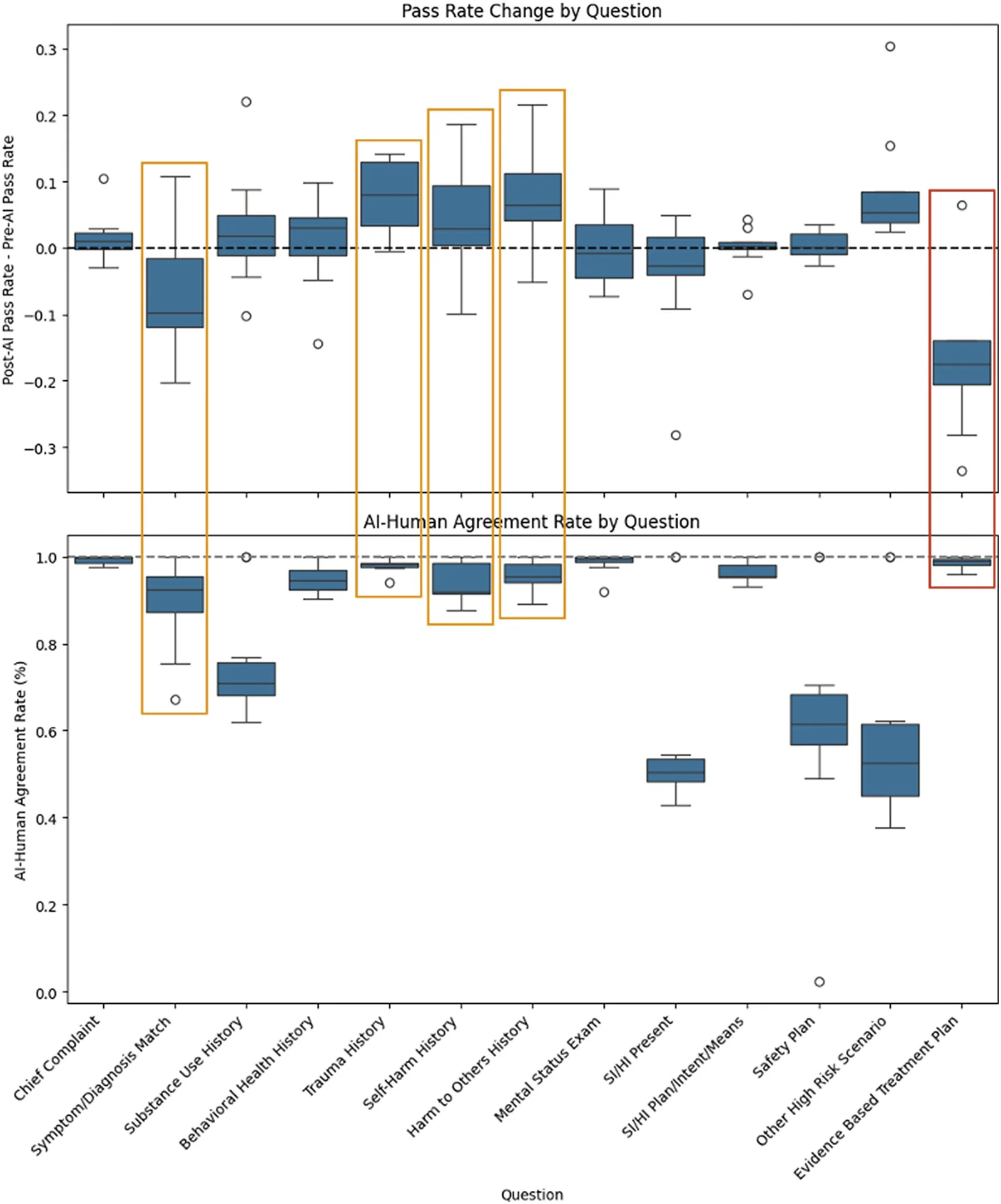
Boxplots of item-level changes in pass rates and auditor agreement with AI predictions. Boxplots illustrate the distribution of changes in pass rates and AI–human agreement across individual rubric items. The upper panel shows the percent change in pass rate between post-AI and pre-AI reviews, centered at 0%. The lower panel shows AI–human agreement with the dotted line at 1.0, representing 100% agreement. Items with medians outside the interquartile range of the overall distribution indicate atypical shifts in auditor behavior. Items highlighted in yellow and red represent items showing both significant pass-rate deviations and high AI–human agreement. AI, artificial intelligence.

### Auditor Speed

Mean review time decreased from 8.77 min (SD: 9.64) pre-AI to 8.06 min (SD: 10.28) post-AI, a difference of 0.71 min per note (95% CI: 0.28–1.14; Welch *t* test, *p* = 0.002), or approximately 71 min saved per 100 notes reviewed (95% CI: 28–114).

## Discussion

In this pre–post evaluation of AI-supported mental health quality auditing, we observed measurable shifts in auditor behavior following the introduction of LLM-generated recommendations into the review workflow. Although the overall increase in pass rates was modest, item-level analyses revealed several items with substantial deviations from typical scoring patterns, particularly those with both large changes in pass rates and high agreement with AI outputs. These patterns suggest that AI recommendations may influence auditor judgment on select items, especially when agreement between auditor and AI recommendations is very high. Importantly, these effects were not attributable to a small subset of auditors but appeared broadly distributed across the reviewer pool, indicating systemwide behavioral shifts rather than isolated outliers. There has been a great deal of interest in applying AI approaches to behavioral health care including treatment decisions, response prediction, protocol adherence, and accelerating of manual processes such as chart auditing. 15 Translating these technologies into current practice remains an emerging process. Prior studies of clinical decision support and AI–human collaboration suggest that automated recommendations influence clinician behavior and can improve performance while also introducing new errors. 16 17 In this study, AI support was associated with a statistically and operationally significant decrease in auditor review time.

These findings underscore a fundamental challenge in AI integration for quality assessment, the absence of an external gold standard for each rubric item. Expert consensus responses used to train and calibrate auditors are the closest available internal reference standard, but they were used for auditor qualification and prompt development rather than for independent adjudication of all study notes. Auditor ratings therefore remain both an operational benchmark and a potential target of AI influence. The observed increase in AI–human agreement presents a critical dilemma. The alignment could reflect a positive outcome, in which AI helps auditors find issues they may have otherwise overlooked, leading to true improvements in rating accuracy. For example, the high AI–human agreement and increased pass rate observed for the Trauma History item could reflect that trauma history may be documented in multiple parts of the note, making it easily missed by auditors. Conversely, increased alignment may reflect automation bias, where auditors defer to AI outputs rather than independently applying rubric criteria. Such deferral would artificially inflate perceived AI performance by reinforcing its own predictions, rather than validating them against an objective standard. If AI guidance encourages auditors to rely too heavily on model recommendations, this could increase the risk of inappropriately passing providers who should have failed, potentially affecting patient safety. It could also lead to inappropriately failing scores and unnecessary consequences for clinicians, such as remediation actions. Without an external gold standard, we cannot determine the direction of correctness, and this uncertainty limits interpretation of improvements or declines in performance metrics in this study.

These findings highlight the nuanced dynamics of human–AI interaction in clinical quality assessment. Even modest changes at the aggregate level can mask meaningful item-level or auditor-level behavioral shifts with operational implications. As AI tools become more deeply integrated into routine quality workflows, monitoring for auditor drift, calibration changes, and disproportionate influence of high-confidence AI outputs will be essential. Future human–AI evaluation studies should consider randomized or crossover designs, blinded adjudication of discordant cases, delayed display of AI recommendations, measurement of cognitive workload, and periodic review of cases with high AI–human agreement but large pass-rate shifts.

The observed decrease in auditor review time represents a direct operational benefit of AI integration, significantly improving efficiency and throughput. Quality reviews supplemented by AI could allow for more comprehensive quality monitoring programs than what is currently feasible for many organizations. The ability to review a substantially greater portion of clinical documentation may provide enhanced empirical insight into associations between clinician practice patterns, documentation, and patient outcomes. While this acceleration is desirable and holds great potential for large-scale quality assurance, it carries potential trade-offs. AI predictions may increase speed by reducing cognitive load, which may foster automation bias and reduced vigilance. Sustained reliance on AI outputs could contribute to professional deskilling if auditors lose their fluency and independent judgment regarding complex or ambiguous cases. Maintaining auditor expertise requires careful design of the human–AI interface so that efficiency gains do not come at the expense of critical engagement with clinical documentation.

This study has several limitations. First, the pre–post design means that changes in post-AI minus pre-AI pass rates may reflect factors other than AI exposure, such as changes in documentation quality, evolving clinical practices, changes in case mix, or modifications to auditor instructions. Auditors also knew that AI support had been newly introduced, which may have led them to scrutinize AI outputs differently than they would in mature routine practice. Although these influences are likely mitigated by the stability of auditing process over the study period and relatively short timeframe, they cannot be excluded. A randomized crossover design with delayed or blinded AI display would better isolate the causal effect of AI exposure and would allow assessment of whether AI effects persist after recommendations are withdrawn. Second, while the patterns found in this study indicate that auditor behavior may be influenced by AI outputs, the direction of correctness cannot be determined from these data. For example, the decreased pass rate for Evidence Based Treatment could reflect AI bias or more accurate scoring if AI recommendations were superior to prior auditor performance. We did not perform an independent adjudicated review of discordant cases comparing pre-AI human ratings, AI recommendations, and post-AI human ratings. Third, the available data did not include detailed demographics for patients, providers, or auditors, such as years since training, tenure at the organization, language, or patient duration of treatment. These factors may influence documentation, auditor interpretation, and generalizability. Fourth, the analysis did not fully model clustering by auditor, note, provider, and rubric item. Finally, we did not directly measure changes in cognitive workload, trust, or decision-making strategies; thus, conclusions about reliance on AI are inferred from behavioral patterns rather than directly observed.

## Conclusion

There is great potential for LLM-based AI tools to enhance the efficiency and rigor of clinical quality review processes by automating routine checks, prioritizing high-risk cases, and identifying subtle documentation patterns that human auditors might miss. In this evaluation, AI-supported auditing was associated with modestly faster reviews and heterogeneous changes in auditor scoring behavior. These findings support cautious implementation: organizations should monitor for auditor drift, item-level calibration changes, automation bias, hallucinated or unsupported rationales, and inconsistent model outputs. Future work should incorporate controlled study designs, validated reference standards, long-term auditor calibration measure, repeated-run reliability testing, and interface designs that preserve independent clinical judgment. Such work will help determine when AI improves quality assessment and when it may inadvertently reshape human behavior.

**Clinical Relevance Statement**

This research demonstrates that while AI integration can significantly streamline the quality auditing process, it can also introduce substantial variability in how individual auditors interpret quality benchmarks. Understanding these behavioral shifts is clinically important for ensuring that LLM-based support enhances, rather than compromises, the objective oversight of outpatient mental health documentation standards and patient safety.

**Multiple-Choice Questions**

1. When introducing machine learning (ML) predictions into psychotherapy quality audits, which risk is most directly raised by very high auditor–ML agreement rates?
  - Reduced statistical power to detect changes in aggregate pass rates.
  - Artificial inflation of apparent ML accuracy due to auditor deference.
  - Increased variability in documentation quality across clinicians.
  - Loss of interrater reliability due to inconsistent rubric interpretation.
  **Correct Answer** : The correct answer is option b. The manuscript highlights that when auditor ratings serve as the primary benchmark for evaluating ML performance, high auditor–ML agreement introduces a critical interpretive challenge. If auditors increasingly align their decisions with ML outputs, this agreement may reflect automation bias rather than true improvement in accuracy. In such cases, ML systems appear more precise, because their predictions are reinforced by human reviewers, not independently validated. This dynamic can artificially inflate perceived ML accuracy, obscuring whether the underlying pass/fail determinations are correct. Without an external gold standard, it becomes impossible to determine whether agreement reflects improved auditing or inappropriate deferral to the ML system. The study emphasizes that this phenomenon has direct operational consequences, including the risk of unnecessary remediation or missed quality deficits.
2. Which finding best supports the conclusion that ML support produced heterogeneous behavioral responses among auditors rather than isolated outlier effects?
  - A modest increase in the overall mean item-level pass rate.
  - A statistically significant reduction in average auditing time.
  - A wide distribution of auditor-level pass-rate changes across individuals.
  - Near-universal ML agreement on selected rubric items.
  **Correct Answer** : The correct answer is option c. Although the aggregate increase in pass rates after ML implementation was small (~2%), the manuscript reports a wide distribution of auditor-level changes ranging from −35 to +35%. This broad spread indicates that auditors responded differently to ML support, with some increasing and others decreasing their pass rates. Further analyses showed that these shifts were not driven by a small number of extreme auditors. Instead, behavioral changes were distributed across the reviewer pool, suggesting a system-level effect rather than isolated deviations. This heterogeneity underscores the importance of monitoring individual auditor calibration when integrating ML tools into quality assurance workflows.
3. Why does the absence of an external gold standard limit interpretation of changes observed after ML implementation?
  - It prevents detection of statistically significant differences between pre- and postperiods.
  - It obscures whether auditor–ML alignment reflects improved accuracy or automation bias.
  - It reduces the generalizability of findings to non-English clinical documentation.
  - It limits the ability to measure changes in auditing speed over time.
  **Correct Answer** : The correct answer is option b. The study emphasizes that auditor ratings currently function as both the training signal and evaluation benchmark for ML-supported quality assessment. Without an independent gold standard, increases or decreases in pass rates and agreement cannot be definitively interpreted as improvements or degradations in accuracy. For example, higher ML agreement could represent correction of systematic human error, or it could indicate auditor overreliance on ML outputs. Because the study design cannot adjudicate between these possibilities, conclusions about correctness must remain cautious. This limitation motivates the authors’ recommendation for future studies incorporating validated external ratings and more controlled designs.

## Declaration of GenAI Use

Authors used Open Evidence and Claude Fable 5.1 AI tools for copy editing this manuscript. The authors reviewed, edited, and approved the content of this manuscript.
